# Acute blindness as a presenting sign of childhood endemic Burkitt’s lymphoma in Cameroon: a case report

**DOI:** 10.1186/s13256-018-1682-3

**Published:** 2018-05-16

**Authors:** Brice N. Vofo, Gaelle V. F. Ngankam, Calypse A. Ngwasiri, Jeannine A. Atem, Leopold N. Aminde

**Affiliations:** 1 Ntam Sub-Divisional Health Centre, Kumba, Cameroon; 2 District Hospital Kumba, Kumba, Cameroon; 3 District Hospital Bamendjou; and Clinical Research Education, Networking & Consultancy, Douala, Cameroon; 4 Baptist General Hospital, Mbingo, Cameroon; 5 Faculty of Medicine, School of Public Health, the University of Queensland, Brisbane, Australia

**Keywords:** Burkitt’s lymphoma, Blindness, Childhood, Ultrasound scan, Case report, Cameroon

## Abstract

**Background:**

Endemic Burkitt’s lymphoma is found predominantly in malaria holoendemic zones, typically in the tropical rain forest of Africa. It usually presents as an extra-nodal tumour in children and young adults with predilection for jaws and soft tissues of the abdomen. Clinical features depend on the primary tumour site, extent of the disease and histologic subtype. Acute blindness as a presentation sign is rare.

**Case presentation:**

A 13 year old African female presented to our facility with a 3 week history of painful abdominal distention, and loss of vision of the left eye. On examination, there was a huge abdominopelvic mass, left breast mass and complete blindness of the left eye associated with mydriatic pupils non-responsive to light. An abdominal ultrasound showed a huge hypoechoic mass arising from the pelvis and protruding into the abdomen. The jaws, teeth and maxilla were all normal. A fine needle aspiration done was negative for malignant cells. A presumptive diagnosis of Burkitt’s lymphoma was made on clinical grounds and abdominal ultrasound findings. The patient was immediately placed on chemotherapy and responded well to treatment with remission of the tumour but persistence of left eye blindness.

**Conclusion:**

Acute blindness can be the presenting sign of Burkitt’s lymphoma in a patient with neither jaw nor maxillary swelling. Primary healthcare providers in low income settings require a high index of suspicion when faced with such atypical presentations. This emphasizes the need for thorough physical examination, and when possible, the clinical utility of ultrasonography for suspicious abdominal masses in the absence of state-of the art diagnostic tools for early diagnosis and treatment, which is critical for survival and to improve quality of life.

## Background

Burkitt’s lymphoma (BL) is a highly aggressive malignant B-Cell non-Hodgkin lymphoma characterized by translocation and deregulation of the c-myc gene on chromosome 8 [1]. It is one of the fastest growing neoplastic human tumour, with a doubling time of about 25 hours and a growth fraction of nearly 100% [2]. In sub-Saharan Africa (SSA), it is the most common childhood tumour that typically affects the jaws and abdomen. Three distinct forms exist epidemiologically: the endemic variant found mainly in equatorial Africa and other tropical locations between latitudes 10 degrees south and north, the sporadic variant, present in North America and Western Europe and the immunodeficiency-associated subtype which is common amongst patients with human immunodeficiency virus (HIV) infection [3].

Occurrence of endemic Burkitt’s lymphoma (eBL) is suggested to correlate with the geographic distribution of endemic malaria and Epstein-Barr virus (EBV) infection. The above relationship has been found in nearly all cases as compared to only 20-30% in the sporadic cases [4] and about 35% in the immunodeficiency-related variant [5]. Its incidence is high among children less than 15 years of age, with special predilection for the abdomen (78%) and head and neck (62%) or both (42%) [6].

Clinical presentation generally depends on the primary tumour site, extent of the disease, and the histologic subtype. Ocular involvement with blindness has been sparingly documented in some African countries [7, 8], and is an infrequent presentation of Burkitt’s lymphoma. To the best of our knowledge, there are almost no published reports from Cameroon. Moreover, data on the treatment and follow-up of patients who presented with acute blindness are very limited. Herein, we present a case of acute blindness due to Burkitt’s lymphoma in a resource limited setting in Cameroon.

## Case presentation

A 13 year old African female presented at our service with a 3 week history of progressive painful abdominal distention and loss of vision of the left eye, associated with anorexia and weight loss. She had no fever, headache, vomiting, bloody urine nor weakness of limbs.

**Fig. 1:**
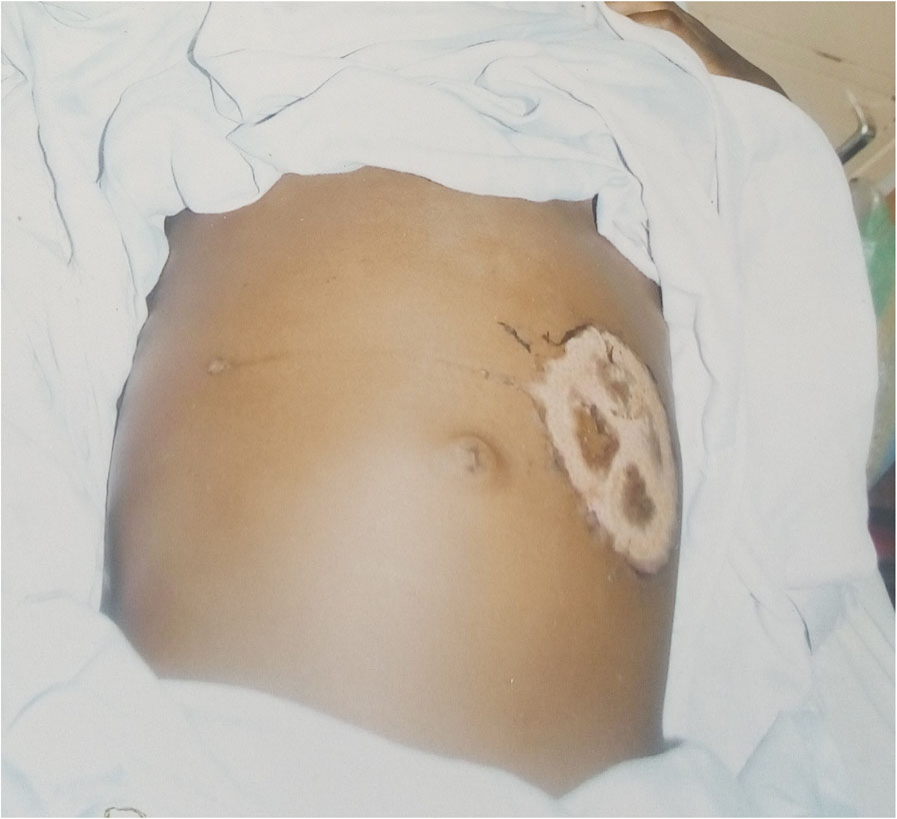
Before chemotherapy (Child with Stage IV Burkitt’s lymphoma)

On physical examination, her vital signs were within normal limits. She weighed 37 kg, and height 147 cm (surface area of 1.2 m2). There was no pallor, no lymph adenopathy and no sign of malnutrition. External evaluation of the left eye revealed complete blindness with a mydriatic pupil that was non- responsive to light. Gastrointestinal examination revealed a circular scarification mark on the abdomen as well as a huge visible abdomino-pelvic mass measuring about 20 cm above the supra-pubic region (Fig. 1). The mass was hard with irregular contours, fixed, moderately tender on palpation and extending to the epigastric region. She had no ascites. The liver, spleen and kidneys were non palpable. A non-tender left breast mass was also palpated, while her mouth, teeth and jaws were normal and likewise the rest of the systemic examination.

A full blood count done revealed lymphocytosis (70%) with normal total white cell count and haemoglobin level of 10.2 mg/dl. She had negative test results for malaria and HIV and her urine analysis was normal. An abdominal ultrasound scan showed a huge hypoechoic abdominopelvic mass, with normal homogenous liver and spleen, while both kidneys had moderate hydronephrosis. Bone marrow aspirate revealed normal trilineage maturation with erythroid hyperplasia and negative for lymphoma or other malignancy. Cerebrospinal fluid (CSF) cytology was negative for malignancy (lymphocytic pleocytosis). Fine needle aspiration of the tumour was done and histopathology analysis of sample showed no malignant cells. A CT scan and MRI were requested but not done due to financial constraints.

Despite the negative bone marrow aspirate results, a presumptive diagnosis of Burkitt’s lymphoma was made based on the clinical picture and abdominal ultrasound findings. The patient was placed on the induction phase of the Burkitt’s lymphoma chemotherapy protocol as adapted from the Malawi 2003 protocol [9].

As per this protocol the patient was due 3 pulses of cyclophosphamide and intrathecal methotrexate and hydrocortisone on days 1, 8 and 15 while maintaining good hydration.

**Fig. 2:**
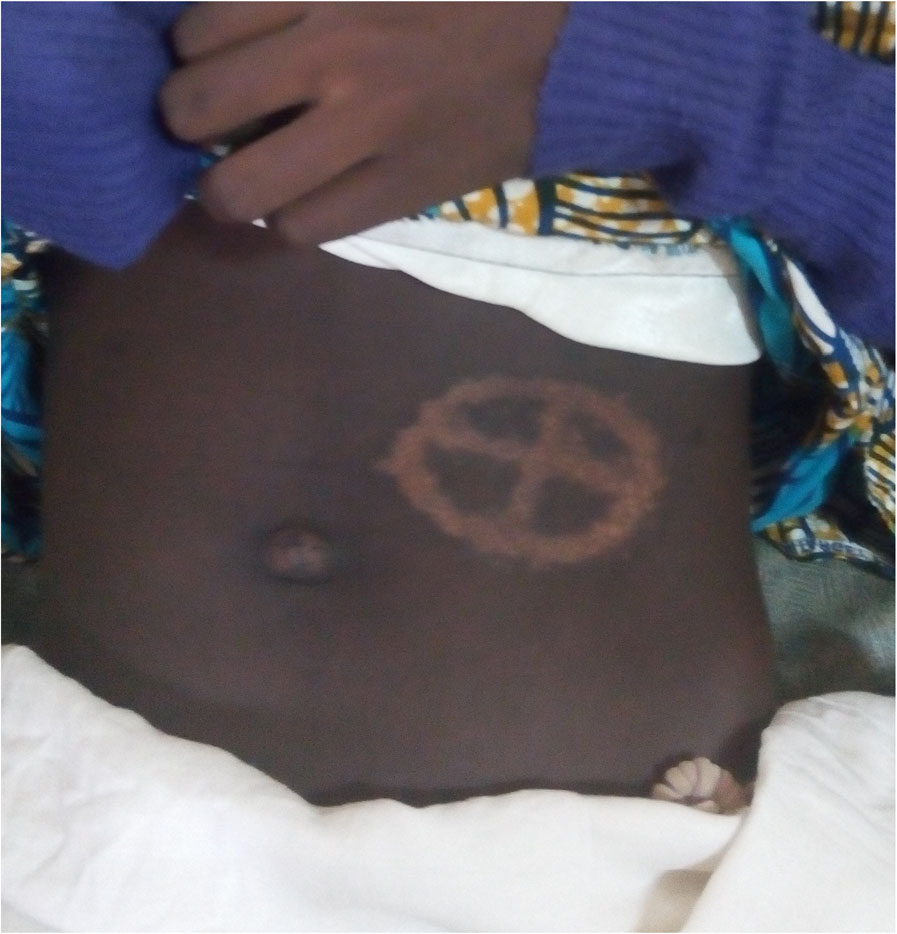
After chemotherapy

Following initiation to treatment, and prior to day 8 (second day of chemotherapy), the breast and abdominal tumours had rapidly regressed. By the end of the induction phase, the left breast mass was no longer palpable though it still appeared bigger than the right whilst the abdomino-pelvic mass had reduced significantly to a small pelvic mass about 6 cm in diameter, non-tender, smooth and firm. The patient however remained blind on the left side. She was then placed on the intensification phase of the chemotherapy protocol. At the end of the chemotherapies, the right breast was now same size with the left, the abdominal mass had completely regressed (Fig. 2) but with persistence of the blindness.

At 6 months follow-up, the primary disease was in remission but there was still blindness of the left eye.

## Discussion

We just presented a 13 year old African girl who presented with a progressive abdominal swelling, breast mass and blindness in the left eye with no jaw swelling. She had a huge, irregular abdominopelvic mass, fixed and mildly tender. A non-tender breast mass was also palpated and she had a fixed mydriatic pupil in the left eye. Amongst the investigations that could be done in this case, only an abdominal ultrasound scan was suggestive of a malignant tumour, as histopathology results of a fine needle aspirate of the mass and bone marrow aspirate were both negative for malignant cells. Despite the negative histopathology results and the uncommon presentation of acute blindness with no jaw swelling in a suspected case of endemic Bukitt’s lymphoma, our patient responded fairly well to the chemotherapy.

The epidemiology of BL shows that the endemic type is mainly confined to equatorial Africa and has a close association with EBV and malaria. Cameroon is a SSA country in the eBL belt and recent evidence shows that the incidence of BL in the North-West region of the country is the second highest in Africa and one of the highest documented worldwide (5.9/100,000) [6]. Studies have shown that constraints on establishing a complete diagnosis and staging, frequency of advanced disease at presentation and high drop-out rates during treatment makes for poorer prognosis [10, 11]. Thus outcome can be improved via early diagnosis and prompt treatment.

Albeit the limited facilities with proper diagnostic tools, basic instruments like the ultrasound scan, increasingly available in limited resource settings like ours, can aid in the diagnosis. The ultrasound scan has been shown to be superior to clinical examination in demonstrating abdominal disease. [12]. More so, abdominal involvement is increasingly reported in our setting due to the augmented use of ultrasound scans and appears to be as frequent as disease of the jaw at presentation [6, 12]. Ocular involvement is not uncommon and orbital disease is usually extra-ocular. However, intra-ocular presentations such as blindness are usually irreversible and rare. This is often associated with orbital lymphoma, in which contiguous spread from neighbouring primary sites in the jaw or maxillary bone is thought to be the main aetiology [13]. This is totally different from adult non-Hodgkins, non-Burkitt’s lymphomas, where intraocular involvement is often a primary disease.

Despite the negative fine needle aspiration results, our patient was placed on treatment for Burkitt’s lymphoma with the understanding that the negative results could be due to inadequate sample collection, which can account for up to 20% of false negatives in our setting, as reported by Marjerrison *et al.* They also reported that out of 95 patients successfully treated for Burkitt’s lymphoma on clinical grounds and ultrasound findings, only 52.7% had positive fine needle aspiration results [12]. In Uganda, Ogwang *et al*. reported that the clinical diagnostic accuracy of Burkitt’s lymphoma was only marginally improved by local pathologist due to inaccuracies [14].

The peculiarity of our case was the presence of intra-ocular involvement (left eye blindness) despite the absence of a jaw or maxillary tumour. This underscores the fact that physicians in endemic zones of Burkitt’s lymphoma should maintain a high index of suspicion in order to avoid delay in diagnosis and treatment of such patients with atypical presentations. Acute blindness is most commonly seen in cases with primary tumours in the skull [7], and a CT scan in this case would have been crucial, though it was not done due to limited financial resources.

## Conclusion

In conclusion, a high index of suspicion among physicians in primary care and rural settings for BL in patients presenting with abdominal mass is critical, bearing in mind that it can progress to loss of vision which could be irreversible. We also highlight the clinical utility of ultrasonography in establishing a diagnosis in the absence of sophisticated diagnostic aids in this settings where the majority of patients seen have limited financial resources to cater for extensive laboratory and imaging investigations. Early diagnosis and treatment remains pivotal to improve survival probabilities and prevent permanent blindness.

## Abbreviations

BL: Burkitt’s lymphoma
eBL: Endemic Burkitt’s lymphoma
EBV: Ebstein-Barr Virus
HIV: Human Immunodeficiency Virus
SSA: Sub-Saharan Africa
